# Use of circulating tumor DNA to guide treatment of primary central nervous system lymphoma: a case report

**DOI:** 10.1093/noajnl/vdab143

**Published:** 2021-09-25

**Authors:** Ka-wai Grace Ho, Tejus Bale, Christian Grommes, Ankush Bhatia, Rachna Malani

**Affiliations:** 1 Department of Neurology, Memorial Sloan Kettering Cancer Center, New York, New York, USA; 2 Department of Neurology, MedStar Georgetown University Hospital, Washington, District of Columbia, USA; 3 Department of Pathology, Memorial Sloan Kettering Cancer Center, New York, New York, USA; 4 Department of Neurology, University of Wisconsin School of Medicine and Public Health, Madison, Wisconsin, USA

**Primary central nervous system lymphoma (PCNSL) is a rare tumor that can be difficult to diagnose as it can mimic other CNS diseases. Tissue biopsy is the gold standard for diagnosis, but this is not always feasible if the lesions are in deep structures of the brain. Tissue samples may also be non-diagnostic if steroids have been administered prior to the procedure. Genomic testing of circulating tumor DNA (ctDNA) in the cerebrospinal fluid is a technique that may provide an alternative method for diagnosis. Prior case reports and studies have shown a correlation between PCNSL and *MYD88* mutation, but patients were treated only in the setting of a diagnostic biopsy. Here we present a case of a patient with inconclusive biopsy results whose CSF ctDNA revealed a *MYD88* mutation suggestive of PCNSL and guided her treatment with high-dose methotrexate**.

## Case Report

**Figure 1.**
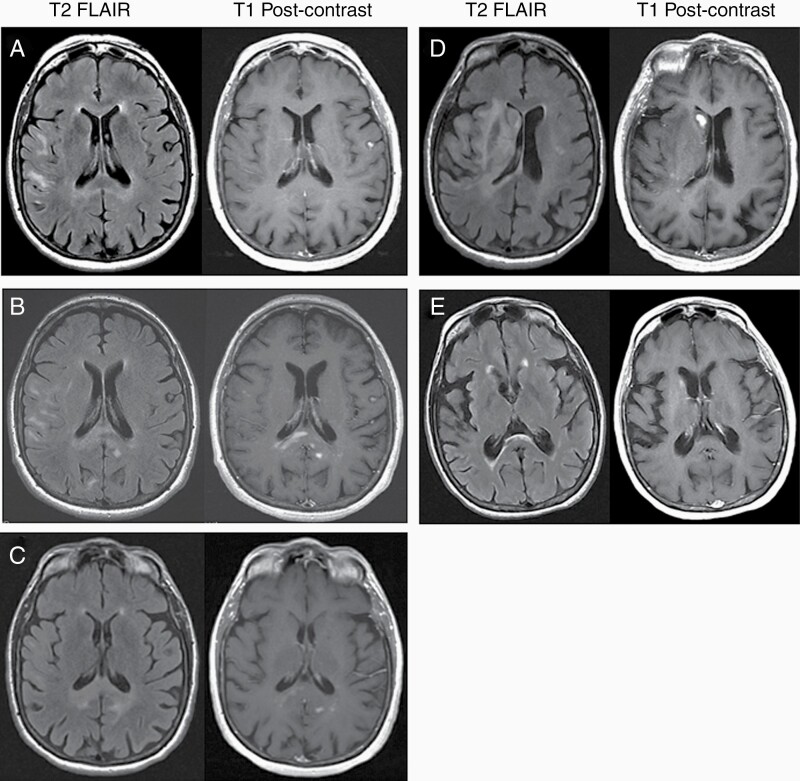
Changes in MRI brain over time. T2 FLAIR precontrast and T1 postcontrast images between initial presentation of symptoms and post-treatment with high-dose methotrexate and cytarabine consolidation. (A) Initial presentation, no contrast-enhancing lesions present. (B) One month after initial presentation, new contrast-enhancing lesions in the corpus callosum. (C) Post high-dose steroid administration, mild reduction in size of enhancing lesions. (D) Just prior to treatment with high-dose methotrexate. (E) Post-treatment with 8 high-dose methotrexate infusions and consolidation with cytarabine.

A 65-year-old woman with metastatic malignant peripheral nerve sheath tumor (MPNST) on active treatment with a novel clinical trial drug and no known germline mutations presented with 6 weeks of nausea, worsening gait, and falls. MRI of the brain revealed nonenhancing white matter abnormalities (Figure 1). Cerebrospinal fluid (CSF) cell count and protein were normal, cytology demonstrated atypical lymphoid cells, and flow cytometry noted an abnormal mature B-cell population that could not be further characterized. CSF viral studies, CSF oligoclonal bands, and both CSF and serum paraneoplastic panels were negative. PET scan of the body showed no evidence of new disease, and ophthalmologic examination was normal. Her clinical trial drug was held, but she worsened symptomatically over the next month with new intermittent diplopia, difficulty holding up her head, and inability to walk. Repeat MRI brain revealed new enhancing lesions, and repeat CSF studies again showed atypical cells with an abnormal B-cell population but normal cell count and protein. The differential diagnosis included an inflammatory or autoimmune process, drug-related process from her MPNST treatment, or a new neoplastic process. Given the deep location of the enhancing lesions, brain biopsy was deferred. She was treated with high-dose methylprednisolone for a presumed autoimmune/inflammatory versus drug-related process.

Over the next several months, her brain MRI showed worsening fluctuations in enhancing and nonenhancing lesions (Figure 1). Brain biopsy of a new enhancing portion revealed gliosis with mild–moderate parenchymal and perivascular mixed inflammatory infiltrate. Genomic testing of the biopsy sample using MSK-IMPACT was nondiagnostic. As there was no evidence of malignancy, she was started on intravenous immunoglobulin (IVIG) and discharged on a prednisone taper. Her symptoms continued to worsen with progressive MRI changes, and she was treated with plasma exchange followed by IVIG and rituximab.

Given her continued lack of improvement, MSK-IMPACT was performed on the CSF and resulted in the discovery of *MYD88*, *CD58*, *HIST1H1C*, *KMT2D*, and *LCK* mutations suggestive of a lymphoid malignancy like primary central nervous system lymphoma (PCNSL). Based on these results, she was treated with 8 doses of rituximab and methotrexate with complete radiographic resolution of disease (Figure 1). She was consolidated with high-dose cytarabine and is now functionally independent.

## Discussion

This case is an example of the importance of CSF ctDNA analysis in the diagnosis of PCNSL in patients who have suspicious lesions in areas that are difficult to biopsy or have nondiagnostic biopsies. PCNSL is a rare type of extranodal non-Hodgkin lymphoma located in the central nervous system that can be difficult to diagnose. Initial clinical presentations and radiographic findings can mimic other disorders such as primary brain tumors, demyelinating diseases, autoimmune or paraneoplastic syndromes, or central nervous system infections.^1^ More than 90% of PCNSL lesions enhance on MRI brain, and the gold standard for diagnosis is via biopsy of a lesion.^2^ In this case, the patient’s treatment with a clinical trial drug with unknown side effects along with her fluctuating symptoms of diplopia and weakness made an autoimmune, inflammatory, or demyelinating process higher on the differential over a second uncommon malignancy with a rare atypical presentation like PCNSL. In addition, her nonenhancing FLAIR hyperintense lesions were in the deep structures of the brain, making biopsy less appealing in the setting of lower clinical suspicion.

Clinicians have attempted to rely on CSF for diagnosis of PCNSL as it is less invasive than biopsy, but neither CSF cell count nor protein levels appear to reliably predict the presence of PCNSL.^2^ In addition, the diagnostic yield in CSF cytology or flow cytometry is often low and do not produce corresponding results. In a prospective study of 123 B-cell lymphoma patients, only 22% of patients had CSF flow cytometry that identified neoplastic B cells, and 8% of patients had conventional CSF cytology with malignant or suspicious cells present.^3^ Other studies have shown that the presence of monoclonal B cells in the CSF may not be diagnostic of clinically significant CNS involvement by lymphoma, as they can also be seen in inflammatory and demyelinating conditions, making it difficult to interpret CSF flow cytometry results.^4^ Atypical cells on cytology can also be observed in patients with infections and autoimmune/inflammatory disorders.^5,6^ Although this patient had CSF cytology with atypical cells and CSF flow cytometry showing an abnormal B-cell population, it was difficult to interpret these results given that an autoimmune/inflammatory process was high on the differential. In this setting, these results alone could not justify a diagnosis of PCNSL and exposure to high-dose methotrexate with its associated side effects including nephrotoxicity, hepatotoxicity, and myelosuppression.^7^

The diagnosis was made by performing next-generation molecular testing on ctDNA in the CSF with MSK-IMPACT, a FDA-authorized DNA sequencing panel targeting over 400 oncogenes and tumor suppressor genes identifying mutations, copy number alterations, and gene fusions.^8^ The presence of *MYD88*, *CD58*, *HIST1H1C*, *KMT2D*, and *LCK* in the patient’s CSF made the molecular diagnosis of PCNSL and guided treatment. Although these mutations have all been linked to hematologic malignancies (Table 1), *MYD88* is of particular interest as it occurs in at least 35% of patients with PCNSL. Fontanilles et al. reported that *MYD88* had a diagnostic sensitivity of 24% for PCNSL but a specificity of 100%.^9^ More recently, Ferreri et al. report a sensitivity of 94% and specificity of 98% for the *MYD88* mutation status and IL-10 levels in the CSF.^10^ Given the specificity of *MYD88* reported in the literature at the time the patient was diagnosed, she was treated with high-dose methotrexate and rituximab even though her brain biopsy was inconclusive.

**Table 1.** MSK-IMPACT Report From CSF ctDNA

Gene	Alternate Name	Type	Alteration	Location	Mean Allele Frequency	Function	Citation
MYD88	None	Missense mutation	L265P (c.794T>C)	Exon 5	28.3%	Adaptor protein	Fontanilles et al.^9^ Ferreri et al.^10^
KMT2D	MLL2, CAGL114, TNRC21m ALR	Frameshift deletion	P1769Lfs*16 (c.5306del)	Exon 22	29.2%	Tumor suppressor and methyltransferase	Ortega-Molina et al.^11^
CD58	LFA3	Frameshift insertion	H44Ffs*20 (c.126_129dupTTTC)	Exon 2	14.1%	Cell surface adhesion molecule expressed in immune cells	Cao et al.^12^
HIST1H1C	H1-2, H1s-1, H1F2, H1c, H1.2	Missense mutation	Q95E (c.283C>G)	Exon 1	14.9%	Histone H1 variant binding to linker DNA between nucleosomes	Okosun et al.^13^
LCK	None	Nonsense mutation	Q87* (c.259C>T)	Exon 4	20.0%	Tyrosine kinase regulating T-cell receptor signaling	Tan et al.^14^

Somatic alterations detected in ctDNA from the patient’s CSF. Overall sequence coverage was 59×.

Although next-generation molecular sequencing allowed for the diagnosis to be made, 1 major drawback is the amount of time it takes to generate the results. Molecular diagnostics can take weeks to months to result whereas frozen pathology from a brain biopsy can confirm the diagnosis before the surgery is finished leading to faster treatment and clinical improvement for the patient. Given how quickly PCNSL patients can deteriorate, rapid diagnosis is paramount to their care. At the time of this diagnosis, MSK-IMPACT took 3 weeks to result, though this has improved over the years. Newer technology such as droplet digital PCR can identify mutations like *MYD88* and can be resulted faster, but they can only be utilized if the clinician is highly suspicious of PCNSL.^15^ In this report, the patient’s diagnosis was unknown in the setting of her MPNST, and the benefit of using a test like MSK-IMPACT is that it targets a wide variety of oncogenes and tumor suppressor genes to help narrow down a diagnosis. This case is an example of how ctDNA can be used to guide treatment of PCNSL in the setting of an inconclusive traditional workup.
